# Screening for persistent high-risk HPV infections may be a valuable screening method for young women; A retrospective cohort study

**DOI:** 10.1371/journal.pone.0206219

**Published:** 2018-10-24

**Authors:** Renée M. F. Ebisch, Pleun J. W. Ketelaars, Wouter M. H. van der Sanden, Channa E. Schmeink, Charlotte H. Lenselink, Albert G. Siebers, Leon F. A. G. Massuger, Willem J. G. Melchers, Ruud L. M. Bekkers

**Affiliations:** 1 Department of Obstetrics and Gynaecology, Radboud university medical center, Nijmegen, the Netherlands; 2 Department of Obstetrics and Gynaecology, Deventer Hospital, Deventer, the Netherlands; 3 Department of Pathology, Radboud university medical center, Nijmegen, the Netherlands; 4 Department of Medical Microbiology, Radboud university medical center, Nijmegen, the Netherlands; Rudjer Boskovic Institute, CROATIA

## Abstract

**Introduction:**

Screening of young women is often discouraged because of the high risk of unnecessary diagnostics or overtreatment. Multiple countries therefore use cytology instead of high risk human papillomavirus (hrHPV)-testing as screening method for young women because of the limited specificity of hrHPV-testing. The objective of this study was to investigate how hrHPV screening before the age of 30, can be used to reduce the future prevalence of high-grade cervical lesions in young women.

**Methods:**

We retrospectively analyzed follow-up data from a cohort study on HPV prevalence in unscreened Dutch women aged 18–29 years. Women performed multiple self-collected cervico-vaginal samples for HPV detection and genotyping. At least one valid cervical pathology result was obtained from 1,018 women. Women were categorized as hrHPV negative, cleared- or persistent hrHPV infection. Anonymized follow-up data for each group was obtained. Composite outcome measures were defined as; normal, low-grade squamous intraepithelial lesion (LSIL) or high-grade squamous intraepithelial lesion (HSIL). The association between prior hrHPV status and cytology and histology outcome was analyzed.

**Results:**

After exclusion, a pathology result was registered for 962 women. The prevalence of HSIL was 19.3% in women with a persistent HPV infection at a younger age. This is significantly higher (p<0,001) compared with the HSIL prevalence of 1.5% in HPV-negative women, and 3.1% (n = 8) in women who cleared the hrHPV infection in the past.

**Conclusion:**

Women with a persistent hrHPV infection in their 20s, show an increased prevalence of HSIL lesions in their early 30s. Screening for persistent hrHPV infections, instead of cytology screening before the age of 30, can be used to reduce the future prevalence of cervical cancer in young women.

## Introduction

Infections with human papillomavirus (HPV) are common; over 80% of the sexually active women have been infected by one or more HPV types at some point in their life [1]. Most HPV infections are transient and clear spontaneously [2]. However, a persistent infection with a high-risk HPV (hrHPV) is known to be a prerequisite for the development of cervical cancer [3,4].

The ris of sexual transmission of HPV generally peaks early in sexual life and declines with higher age [5]. Therefore, younger women more frequently test positive for hrHPV than women over 30 years of age [6]. The majority of infections does not lead to cervical cancer at such a young age. HrHPV screening therefore is highly sensitive for detection of CIN2+, but holds a limited specificity, resulting in a high risk of overtreatment. This is especially the case for young women because of a high rate of transient infections in these women. To mitigate this, many countries delay screening start until the age of 25 or 30, or use cytology as screening method for young women [7,8].

Cytology screening has limited sensitivity, lacks the advantage of using self-sampling as primary screening method, and it is known that self-sampling increases participation of non-responders to cervical cancer screening [9]. The addition of self-sampling in the Netherlands is expected to increase detection of cervical cancer with 10% and to decrease mortality with approximately 8%.

From 1996 to 2016 Dutch women were screened in a cytology-based organized 5-yearly cervical cancer screening program, from the age of 30 to 60. The Dutch National Cancer Registry shows that in 2016 6.1% of all cervical cancers are diagnosed in women aged between 20 and 29 years of age and another 12.9% is diagnosed in women aged between 30 and 35 years. So, 19.0% of all cervical cancers are detected before the age of 35. Starting cytology screening at the age of 30 probably detects cancers, but did not prevent these cases of cervical cancer. A hrHPV-based cervical cancer screening strategy with cytology triage for women under 30 could therefore potentially prevent these cancers by detecting hrHPV infections and cervical intraepithelial neoplasia (CIN) lesions before progression to cancer [10].

The objective of this study was to investigate how hrHPV screening before the age of 30, can be used to reduce the development of cervical cancer in young women.

## Materials and methods

In this cohort study, we retrospectively analyzed follow-up data from a large prospective cohort study on HPV prevalence, incidence and clearance in women under the age of 30 which was performed in the Netherlands in 2007 [11,12]. In the original study, unscreened women aged 18–29 were recruited, using different advertisements, as well as active recruitment sites, and posters at general practices in the city regions of Arnhem, Nijmegen, and Den Bosch, the Netherlands. Furthermore, advertisement on the internet were used, which were accessible in the whole of the Netherlands. In total, 2,065 unscreened women were included. Women performed a 3-monthly self-collected cervico-vaginal sample for 12 months. All women received a self-sample kit and questionnaires by mail and performed the cervico-vaginal self-sample in the privacy of their own home. Self-samples were tested for the presence of HPV with full genital HPV genotyping. Polymerase chain reaction (PCR)-based hrHPV testing on self-samples has been shown equally sensitive compared with clinician-based samples [13]. When women were hrHPV-positive after 12 months, another 12 months’ follow-up with 6-monthly HPV testing was offered. If women were hrHPV positive at the end of 24 months (t = 24 months), a clinician-taken smear for cytology testing was advised. Patient characteristics of this group of 2,065 women are previously described [11,12]. The self-samples were tested for the presence of HPV by using the highly sensitive SPF_10_-DEIA, and genotyping of HPV positive samples was performed with the SPF_10_-LiPA [14].

From the total cohort of 2,065 women, 1,333 were aged over 30 at June 1st 2015 and were included in this study, as they at that point had been invited for the first screening round of the national cervical cancer screening program in the Netherlands. Women who were only tested once (n = 371) because they chose not to participate any further in the study from Lenselink et al., and Schmeink et al, were excluded because HPV persistence or clearance could not be determined with one single test [11,12]. Clearance was defined as a final negative hrHPV test. Women with two or more hrHPV negative test results, and no hrHPV positive results were classified as ‘hrHPV negative’. Women with one or more hrHPV positive test result followed by only hrHPV negative test results were classified as ‘cleared infection’. Women who still tested hrHPV positive at the end of the 24-month period were classified as ‘persistent infection’. The ‘persistent infection’ and ‘cleared infection’ groups were subcategorized according to the presence or absence of HPV16 or HPV18, independent of the presence of the other hrHPV genotypes (i.e., 31, 33, 35, 39, 45, 51, 52, 53, 56, 58, 59, 66, 68 and 73) detected by de SPF10-LiPA.

Women were categorized according to their HPV test result. Anonymized 8-year follow-up data for each group was obtained from the Dutch registry of histopathology and cytopathology with nationwide coverage (PALGA, Houten, the Netherlands). This follow-up period includes time from the final HPV-test point (t = 24 months) until the PALGA search eight years later Date of birth and first four letters of the surname were used as a personal identifier. For all women, cervical cytology and histology results registered up to June 1^st^ 2015 were collected. Results were anonymized by assigning a random study-number. This anonymization was obliged by the Dutch registry of histopathology and cytopathology (PALGA).

If a histology result was available; the most severe histology result was used as outcome measure, otherwise the most severe cytology result was used as outcome measure. The Dutch CISOE-A classification system was used to report the test results for the cervical smears, which can easily be translated into the Bethesda nomenclature [15]. Cervical cytology samples were listed as ASC-US when they showed atypical squamous cells or squamous metaplasia, atypical repair, or atypical glandular cells (scored as S2-3, O3 or E3 in the CISOE-A classification), and listed as LSIL, when they showed mild dyskaryosis of the squamous epithelium, mildly atypical endometrium, or mildly-moderately atypical endocervical epithelium (scored as S4, O4 or E4-5). Cervical smears that showed moderate dyskaryosis of squamous epithelium or worse, moderately atypical endometrium or worse, or severe atypical endocervical epithelium or worse (scored as S5-9, O5-9 or E6-9), were registered as HSIL [15].

The first six months of follow-up after the final sample (t = 24 + 6 months) for the initial prospective study were censored from analysis because cervical cytology and histology results from these first months of follow-up were most likely results from the advised clinician-taken sample. These would possibly not have been detected without the clinician-taken sample advised by the study, and therefore excluded to minimize bias because of this advice Women with HSIL or cervical cancer results in these first six months were completely excluded from follow-up as treatment of these lesions most likely affected the natural course of events. Furthermore, women for whom no valid cytology or histology result was registered after censoring of the first six months were excluded, as well as women with an uncertain identity. The identity was uncertain when the woman’s first name from the study database did not match the first name from the PALGA database, because it is possible that these are two different women with the same surname and date of birth.

Composite outcome measures were obtained, defined as; negative for intraepithelial lesion and malignancy (NILM), including normal histology and normal cytology results; low-grade squamous intraepithelial lesions (LSIL), including CIN grade 1 histology and atypical cells of undetermined significance (ASC-US) and LSIL cytology; and high-grade squamous intraepithelial lesions (HSIL), including histology results of CIN grade 2 or worse and HSIL cytology.

The association between prior hrHPV status and cytology and histology outcome was analyzed. Also, the duration of the hrHPV-infection until clearance was analyzed in regard to the outcome. Significance was calculated using the Fisher’s Exact test, with a p<0.05 threshold for significance. All statistical analyses were performed using SPSS version 22.0 (IBM, Armonk NY).

The study was approved by the scientific committee of PALGA. The study was exempt from institutional review board approval because data were gathered retrospectively and analyzed anonymously.

## Results

**Fig 1:**
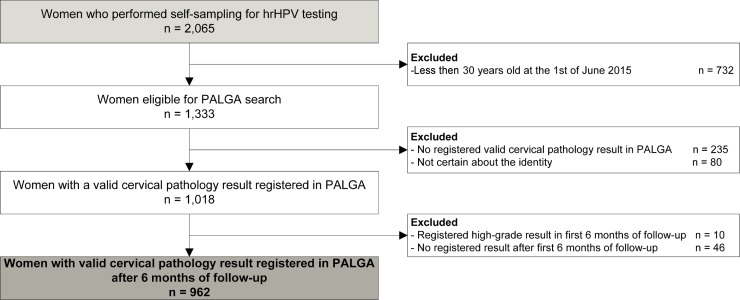
Histology and cytology follow-up results in regard to groups based on previous hrHPV results. hrHPV: high-risk human papillomavirus, HSIL: high-grade squamous intraepithelial lesion, LSIL: low-grade squamous intraepithelial lesion. Differences marked with an * are statistically significant with a p value <0.05. Note that the thin columns are subcategories of the overall categories.

From the cohort of 1,333 women, one or more valid cervical pathology result was obtained from 1,018 women, resulting in a follow-up rate of 76%. For 235 women, no valid cervical pathology result was registered, and for 80 women the identity was uncertain. Forty-six women had no registered pathology result after censoring the first six months of follow-up, and were excluded. For 10 women, a high-grade result was registered in the first six months of follow-up after the final sample was taken (t = 24 + 6 months), and these were also excluded from further follow-up. This resulted in a group of 962 (72.2%) women aged over 30 for which follow-up data was available (**Fig 1**). This resulted in an approximated 7,215 person-years of follow-up. These 962 women were subcategorized in groups according to their previous hrHPV results, as described. Of the total cohort, 591 women (61.4%) were hrHPV negative, 257 (26.7%) showed a cleared hrHPV infection, and 114 (11.9%) had a persistent hrHPV infection (**Table 1**).

**Table 1:** Characteristics of groups of women in regard to groups based on previous hrHPV results.

Groups based on previous hrHPV results	N	% (95% CI)
**hrHPV negative**	591	61.4 (58.3–64.5)
**Cleared hrHPV infection**	257	26.7 (24.0–29.6)
Cleared HPV16/18 infection	78	8.1 (6.8–9.7)
Cleared other hrHPV infection	179	18.6 (16.3–21.2)
**Persistent hrHPV infection**	114	11.9 (10.0–14.1)
Persistent HPV16/18 infection	42	4.4 (3.2–5.9)
Persistent other hrHPV infection	72	7.5 (6.0–9.3)
**Total**	**962**	**100**

CI: confidence interval; hrHPV: high-risk human papillomavirus. Other hrHPV includes HPV types 31, 33, 35, 39, 45, 51, 52, 53, 56, 58, 59, 66, 68 and 73.

During follow-up, 841 (87.4%) women had a normal cervical smear or normal cervical histology, 82 (8.5%) women had LSIL cytology or histology, and 39 (4.1%) women had HSIL cervical histology or cytology results registered in the PALGA database (**Table 2**). The prevalence of HSIL in follow-up was 19.3% for women with a 24-month persistent hrHPV infection. This is significantly higher (p<0.001) compared with the 1.5%, HSIL prevalence in hrHPV-negative women, as well as the 3.1% HSIL prevalence in women with a cleared hrHPV infection. In HPV16/18 persistent infections the HSIL prevalence was highest at 28.6%. Persistent infections with the other hrHPV types showed a HSIL prevalence of 13.9%, which was not significantly different from the persistent HPV16/18 group (p = 0.84). Persistent infections also show the highest percentage of LSIL follow-up, although with a lower percentage of LSIL in HPV16/18 infections with 7.1%, compared with an 18.0% incidence of LSIL in persistent other hrHPV infections. Of the hrHPV negative group, one woman was diagnosed with micro invasive cervical cancer. In total, 17 women were diagnosed with CIN grade 3, and 21 women were diagnosed with CIN grade 2 (**Table 3**). As may be expected, hrHPV negative women showed the lowest future prevalence of HSIL (1.5%). The highest future prevalence was estimated for women who still showed a positive hrHPV test after 12 months, with a HSIL prevalence of 19.5% during follow-up. For women who cleared their infection within 12 months, the HSIL future presence was significantly lower (p<0,001) with a HSIL prevalence of 3.1% (**Table 4**). Differences between HPV16/18 and other hrHPV types were not studied as groups were too small.

**Table 2:** Histology and cytology follow-up results in regard to groups based on previous hrHPV results.

Groups based on previous hrHPV results	Normal		LSIL		HSIL		Total
	N	% (95% CI)	N	% (95% CI)	N	% (95% CI)	*N*
**hrHPV negative**	537	90.9 (88.3–92.9)	45	7.6 (6.0–9.6)	9	1.5 (0.8–2.9)	*591*
**Cleared hrHPV infection**	228	88.7 (84.2–92.1)	21	8.2 (5.4–12.2)	8	3.1 (1.5–6.1)	*257*
Cleared HPV16/18 infection	73	93.6 (85.5–97.6)	4	5.1 (1.6–12.9)	1	1.3 (0.0–7.6)	*78*
Cleared other hrHPV infection	155	86.6 (80.8–90.9)	17	9.5 (5.9–14.8)	7	3.9 (0.18–8.0)	*179*
**Persistent hrHPV infection**	76	66.7 (57.6–74.7)	16	14.0 (8.7–21.7)	22	19.3 (13.0–27.6)	*114*
Persistent HPV16/18 infection	27	64.3 (49.1–77.1)	3	7.1 (1.8–19.7)	12	28.6 (17.1–43.7)	*42*
Persistent other hrHPV infection	49	68.1 (56.6–77.7)	13	18.0 (10.7–28.6)	10	13.9 (7.5–23.9)	*72*
**Total**	**841**	**87.4 (85.2–89.4)**	**82**	**8.5 (6.9–10.5)**	**39**	**4.1 (0.3–0.6)**	***962 (100)***

Note that numbers and percentages of the subgroups in cleared and persistent infections are added up in the total group of cleared and persistent infections, and therefore do not add up to the total columns. CI: confidence interval; hrHPV: high-risk human papillomavirus; HSIL: high-grade squamous intraepithelial lesion; LSIL: low-grade squamous intraepithelial lesion. Other hrHPV includes HPV types 31, 33, 35, 39, 45, 51, 52, 53, 56, 58, 59, 66, 68 and 73.

**Table 3:** HSIL endpoints in regard to groups based on previous hrHPV results.

Groups Based on previous HPV results	CIN2	CIN3	Invasive carcinoma	Total N	% (95% CI)
**hrHPV negative**	5	3	1	9	23.1 (12.4–38.5)
**Cleared hrHPV infection**	4	4	0	8	20.5 (10.5–35.8)
Cleared HPV16/18 infection	0	1	0	1	2.6 (0.0–14.4)
Cleared other hrHPV infection	4	3	0	7	18.0 (8.7–33.0)
**Persistent hrHPV infection**	12	10	0	22	56.4 (41.0–70.7)
Persistent HPV16/18 infection	6	6	0	12	30.8 (18.5–46.5)
Persistent other hrHPV infection	6	4	0	10	25.6 (14.4–41.2)
**Total N (%)** 95% CI	**21 (53.9)** 38.6–68.4	**17 (43.6)** 29.3–59.0	**1 (2.6)** (0.0–14.4)	**39 (100)**	**100**

Note that numbers and percentages of the subgroups in cleared and persistent infections are added up in the total group of cleared and persistent infections, and therefore do not add up to the total columns. CI: confidence interval; hrHPV: high-risk human papillomavirus; HSIL: high-grade squamous intraepithelial lesion; LSIL: low-grade squamous intraepithelial lesion. Other hrHPV includes HPV types 31, 33, 35, 39, 45, 51, 52, 53, 56, 58, 59, 66, 68 and 73.

**Table 4:** Follow-up results in regard to frequency of subsequent hrHPV detection.

	Normal	% (95% CI)	LSIL	% (95% CI)	HSIL	% (95% CI)	Total (%)
**hrHPV negative**	537	90.9 (88.3–92.9)	45	7.6 (5.7–10.1)	9	1.5 (0.8–2.9)	591 (100)
1-4x hrHPV positive (0–9 months)*	230	89.1 (84.7–92.4)	20	7.8 (5.0–11.7)	8	3.1 (1.5–6.1)	258 (100)
5-7x hrHPV positive (12–24 months)**	74	65.5 (56.3–73.6)	17	15.0 (9.5–22.9)	22	19.5 (13.2–30.8)	113 (100)
**Total**	**841**		**82**		**39**		**962**

*1–4 hrHPV infections represent infections that cleared within 3–12 months.

**5–7 hrHPV infections represent infections that did not clear within 12–24 months. CI: confidence interval; hrHPV: high-risk human papillomavirus; HSIL: high-grade squamous intraepithelial lesion; LSIL: low-grade squamous intraepithelial lesion

## Discussion

This cohort study shows a significant increase in prevalence of HSIL in the early 30s of women who had a persistent hrHPV in their 20s. Especially women with a hrHPV infection still present after 12 months showed a significantly higher future prevalence of HSIL, compared with women with a hrHPV infection that was cleared within 12 months.

In line with our results, previous studies have shown that women with a persistent hrHPV infection have a significantly higher risk of developing high-grade CIN lesions compared to those who cleared their infection [16,17]. Data from this study adds the increased prevalence of HSIL in the future for these young women. HPV prevalence and persistence have especially been shown to be high among sexually active young women; with a HPV prevalence up to 54%, of which 34% was a persistent infection [18]. It is however known that these infections only rarely cause cervical cancer at such young age [10], and that overtreatment especially at young age is undesirable because of the risk of cervical insufficiency in future pregnancies [19]. Therefore, most countries use cytology screening for women between the age of 20 and 30 years, instead of hrHPV-based screening. In the Netherlands screening starts at the age of 30, and younger women are not screened at all, in order to prevent overtreatment of young women. A study by van der Aa et al. in 2008 concluded that lowering the age for cervical cancer screening was not useful at that time because of a stable incidence and mortality rate for cervical cancer among women younger than 30 years [20]. However, the cervical cancer rate in women under 35 in 2005 was 14.6%, and shows an increasing trend with 19.0% in 2016. Disadvantages of screening might therefore be proportionate compared with the advantages of screening women in their 20s.

Various countries differ in the organization of their cervical cancer screening program; in terms of type of screening test, invitation methodology, population-based screening or opportunistic screening, target population, screening intervals, but also in age of starting cervical cancer screening. In the Netherlands and Finland screening is offered by the government from the age of 30 years with a 5-yearly schedule. Belgium, France, Australia, the UK and Italy start earlier and invite women every 3–5 years starting at their 25th birthday. Sweden starts at the age of 23, and Germany, Canada, and the USA start even earlier at 20 or 21 years old and use a 1–5 yearly schedule. Some countries already use a hrHPV-based screening program, others still use the Pap-test as primary screening method, and others combine them and use co-testing. However, the cervical cancer incidence in these countries is not inevitably linked to the start of screening or screening interval [21]. Germany offers the most screening rounds, but the cervical cancer incidence is lowest in Finland where women are screened 5-yearly from the age of 30. Participation in the screening program is known to be important in lowering cervical cancer incidence, and young age is a risk factor for non-participation [21]. Participation to screening programs may be improved by offering self-sampling for hrHPV testing [9], which has been shown to be equally sensitive to clinician-taken samples for hrHPV testing and may be an attractive option for screening young women [13].

In the Netherlands in 2016, 19.0% of all cervical cancers was diagnosed in women aged between 20 and 35 years. The majority of these cancers may potentially be avoided if a persistent infection in a girls’ 20s was detected and present CIN lesions were treated in time. For example, it could be considered to start hrHPV-based screening for all women at the age of 20 or 25. Data from this study show that a HSIL lesion is only present in 3.1% of women with a hrHPV infection which is cleared within 12 months, and no carcinomas were found in this group. The prevalence of HSIL lesions is much higher at 19.5% when women still test hrHPV positive 12 months after their first positive hrHPV test. Performing cytology triage after one single hrHPV positive test may therefore result in referral of too many young women for colposcopic examination with possibly unnecessary diagnostics and unnecessary treatment. However, there are no studies comparing cytology triage after one single positive hrHPV test and performing triage after two consecutive positive hrHPV tests, so it is unknown which strategy would be more effective and cost saving.

It may be valuable to individualize screening for young women. Young women with one hrHPV positive result can then monitored with a watchful waiting policy to see whether the infection will clear within 12 months. When these infections do not clear within the 12 months, cytology triage should be performed to detect the presence of CIN lesions in need of treatment, to prevent development of cervical cancer before the age of 30 years.

Contrary to the increased future prevalence of HSIL lesions in young women with a persistent hrHPV infection, this study shows that women who are hrHPV negative in their early adulthood show low prevalence (1.5%) of being diagnosed with HSIL lesions in the next eight years of follow-up. Women who are hrHPV negative before the age of 30, might even benefit from longer screening intervals. However, further studies should be performed to see if extending screening intervals would be safe. Also, the discovery of one micro-invasive squamous cell carcinoma in the hrHPV negative group of this study might contradict this suggestion. Because all follow-up data was anonymized we could not identify other baseline characteristics, and how many hrHPV negative tests preceded this carcinoma. These were at least 2 negative tests because all women with two or more hrHPV negative test results and no hrHPV positive results were scored in this group. Also, we could not identify when these tests were hrHPV negative, or if the carcinoma was hrHPV positive or negative. As follow-up in this study was 8 years, this carcinoma may also have developed quickly.

From the total group of 1,333 eligible women, at least 1,018 (76.4%) women had a cervical smear taken. This percentage is high compared to cervical cancer screening participation in young women in the Netherlands which ranges between 50–60% in women aged 30–35 years [22]. The knowledge on hrHPV obtained by participating in the study or knowledge of their hrHPV status may have affected women in their choice of having a cervical smear taken in the first cervical cancer screening round. Other potential sources of bias in this study could be that the SPF is a highly sensitive surveillance assay, not a clinically validated diagnostic assay, which could possibly result in detection of clinically irrelevant infections. Furthermore, there is the possibility that an infection classified as persistent is not truly a persistent infection, but could also be a re-infection with the same or another hrHPV type. Persistence in this study was purely based on the presence of HPV16/18 or any other hrHPV type detected. This dichotomy was chosen because of privacy of anonymized data. A re-infection however might still indicate increased susceptibility for hrHPV and additional cytology triage might be needed. Also, ASCUS pap-smears were categorized as LSIL in this study. In fact, these two results are not directly comparable, which could also have caused potential bias with increased numbers of LSIL in different groups. This was however done in all groups, so potential bias would be present in all groups equally. Furthermore, the first six months of follow-up after the final sample for the initial prospective study (t = 24 + 6 months),) were censored from analysis, and 10 women with HSIL or cervical cancer results in these first six months were excluded because treatment of these lesions most likely affected the natural follow-up. Censoring of these first six months and exclusion of 10 women with HSIL or cervical cancer might have caused bias which could cause an underestimation of our results.

From 2009 on, prophylactic hrHPV vaccination with the bivalent vaccine is offered to girls in the Netherlands, with a coverage-rate of 61% in 2016 [23]. These vaccinated women will first enter the organized screening program at the age of 30, which will be the case in 2023. Screening strategies for women under the age of 30 will therefore still be beneficial as not all girls are vaccinated, and it is unknown to which extent vaccinated girls will be protected for cervical cancer caused by hrHPV types other than HPV16 and HPV18. These differences in risk-estimates for an hrHPV infection with HPV16 or HPV18 might be a reason for individualizing screening in the future. Earlier research has shown that young women aged between 21 and 30 in the United States who had not initiated HPV vaccination were also less likely to have a Pap test, compared to women who initiated vaccination [24]. The option of hrHPV self-sampling in the privacy of their own homes might persuade these young women to indeed participate in cervical cancer screening [9].

Multiple studies have shown the value of HPV16/18 genotyping in triage of hrHPV positive women [25,26]. This study also shows the highest HSIL prevalence in the future for women with a persistent HPV16/18 infection, which is twice as high compared with the HSIL prevalence in the future in women with a persistent infection with one of the other hrHPV types. This confirms that HPV16/18 genotyping may indeed be useful in individualized screening, triage, and follow-up strategies of hrHPV positive women. However, the numbers in this study are too small to draw specific conclusions in this group of young women.

## Conclusions

This study shows that women with a persistent hrHPV infection in their 20s, show an increased prevalence of a HSIL lesion in their early 30s. Screening for persistent hrHPV infections before the age of 30, instead of cytology screening, can be used to reduce the prevalence of cervical cancer in young women.
